# Sex/gender conservatively, progressively, and in-between: The politics of ambiguity in Pajtim Statovci’s *My Cat Yugoslavia*

**DOI:** 10.12688/openreseurope.20789.2

**Published:** 2026-01-18

**Authors:** Eric Bergman

**Affiliations:** 1 Faculty of Humanities and Social Sciences, University of Zagreb, Zagreb, 10000, Croatia

**Keywords:** Post-Yugoslav literature, sex/gender, in-betweenness, structural dialogism, narrative gap, patriarchy

## Abstract

This paper examines the formal ways in which textual ambiguity can lead to differing and legitimate readings concerning sex/gender (Fausto-Sterling 2012) as a political issue. Pajtim Statovci’s novel *My Cat Yugoslavia* (2017 [2014]) tells the story of Bekim, whose parents moved the family from Kosovo to Finland in the 1990s due to war. The story is narrated by Bekim and his mother Emine in turn, which, I argue, is a structural dialogism that destabilizes each of the narrators’ perspectives. Bekim desires men, which, in the conservative milieu of Albanian Kosovo, is almost unimaginable, although Bekim is now a Finn, albeit marked by in-betweenness. As such, Bekim’s initial description of the male sexual act shapes the way in which his mother’s description of her marriage in Kosovo in the 1980s is read. I argue that this layered narration is a method of destabilizing gendered and sexual norms and questioning the political import of ‘categorical’ and ‘practical’ identities (Hogan 2018). Furthermore, I suggest that the ambiguity concerning sex/gender resulting from the alternating narrative form allows for, and in fact invites, various readings based on readers’ politics. As such, textual ambiguity allows for both ‘progressive’ and ‘conservative’ readings of sex/gender in the novel, presented here heursitically to imply a multiplicity of other readings, and the ambiguity can be termed a narrative gap (Caracciolo and Guédon 2017; Iser 1978) that is filled in by readers. I also argue that the issue of Bekim’s ambiguous sex/gender positioning can be extended to highlight the consequential yet perhaps less-emphasized positioning of Emine as a woman. *My Cat Yugoslavia* is not only an intervention in queer politics, but also in the politics of feminism and migration.

People tend to have set opinions about how one’s sex should manifest in the world. These opinions are often not based on cutting-edge scientific research but are rather intuitive—based on the hegemonic norms of one’s upbringing and environment. The fact that there are ‘males’ and ‘females’ is hardly a contentious statement; that this category (sex) should correlate with behavior (gender) will begin to see a larger division in opinions. This paper examines a text, Pajtim Statovci’s (2017) *My Cat Yugoslavia*, that supports various opinions concerning the link between sex and gender. The ambiguity of the text results in a political situation in which contradictory positions are infused into a single textual artifact. Statovci commented on his preference for leaving meaning in his texts open ^1^. This ambiguity leaves a narrative gap that readers will fill based on their worldviews (see, e.g., 2– 6). The results of textual ambiguity are thus manifold, and can have interesting and perhaps unforeseen political outcomes.

Sex is a fundamental category by which humans are parsed, and gender is normatively correlated with sex ^7^. The fundamental categorical division of male/female, largely consistent across the cultures of the world ( 7 on page 40; see, however, e.g., 8 on page 1053), is of course open to any number of variations (see, e.g. 9) however, based on sexual dimorphism ( 7 on pages 6–8, 12–13), these remain exceptions to the general rule. As social constructs, however, conceptions of gender change over time and show variation but, through sociocultural norms, are often tied to sex in a straightforward manner in any given context. As such, the alignment of sex and gender, or sex/gender, ^2^ (i.e., a male [sex] will be masculine [gender]) is the normative categorical nexus *par excellence*, so engrained in our everyday lives, institutions, laws, codes, and traditions as to remain, in many situations, unquestioned.

Scholarship, from feminism and gender studies to queer studies and beyond, has been on a mission to deconstruct the sex/gender categorical nexus and, from an academic standpoint, has largely succeeded (I am thinking here of post-structuralism generally and Judith Butler’s work as an exemplar). However, outside academia, sex/gender remains an intuitive way to categorize the world. The fact remains that much of the world holds on to a deeply engrained ‘folk’ ^7^ understanding of the correlation between sex and gender.

*My Cat Yugoslavia* presents us with a fascinating case study by which to attempt to untangle the tensions arising from two worldviews concerning sex/gender—here and below in reference to its role beyond reproduction—which are here termed, in a simplified heuristic, ‘conservative’ and ‘progressive.’ ^3^ The conservative worldview posits that there are inherent differences between the sexes (see 9) and that a stable and normative correlation between sex and gender is desirable; while exceptions occur, these may be suspect. The progressive worldview holds that though sex/gender may be normative for most people in most walks of life, there is no inherent correlation (or vanishingly little) between sex and gender; the imposed normative correlation between sex and gender is undesirable because it is false. Sex/gender should rather be understood as existing as a spectrum, and hence as multiple, rather than as a simple male/female binary. There are, of course, myriad ways to think about sex/gender that fall outside of these generalizations, but, based on the novel at hand, I will show that the conservative/progressive binary is a useful lens by which to read the novel.

The question of conservative and progressive understandings of sex/gender can be asked because the text itself opens, largely through ambiguity, both worldviews as possible answers to the character-narrator Bekim’s troubles. Conservatively, it can be said that Bekim’s in-between sex/gender positionality, which is to say being a male but behaving in feminine ways, stands in the way of a coherent or ‘healthy’ identity. By a ‘healthy’ identity I here refer to what Patrick Colm Hogan ( 7 on page 7) calls “folk psychology,” which is based on intuitive interpretation aligned with consensus social norms. Hogan writes, “These intuitive theories orient our observations in certain ways and incline us to draw certain sorts of causal inference” ( 7 on page 7) based on “gross, external features” ( 7 on page 8). As such, the determination of a ‘healthy identity’ is in the eye of the beholder, which in this case is the reader who bases such judgments on evidence from the text and prior personal stances. The conservative reading could be supported by Bekim’s lack of agency, angst and depression, and his unreliability. Bekim remains troubled in relation to sex/gender, which, a conservative reading would suggest, has to do with his inability to align himself with sex/gender norms by, for example, making peace with the problematic masculinity of his father. ^4^

The progressive reading would agree that Bekim’s positionality is in-between sex/gender norms, but with different results, as well as reasons to explain why this may be so. The progressive reading posits that sex/gender norms are, in fact, a performance, and Bekim’s ability to subvert these norms points to a liberating move. Bekim’s sex/gender norms are inherited from his family—conservative, Muslim, from rural Kosovo—which he rejects having grown up in Finland. Bekim’s profound hatred of his father is based on his rejection of patriarchy and harmful masculinity, which has caused him trauma via the father’s abuse and control and brought him to associate with the femininity of his mother, who also suffered at the hands of the father. Also, the novel’s divergence from a realist setting with talking animals and human-animal sexual relationships can shed light on Bekim’s particular queerness. This ‘fantastic’ element can be taken as a mockery, as it were, of a delimited definition of sex/gender and sexuality through highlighting the surrealness of sex/gender identity politics. If the conservative reading takes ‘men who have sex with men’ (MSM) as abnormal, what about MSC (men who have sex with cats) or MSS (men who have sex with snakes)?

The conservative and progressive readings can be understood as the text being directed towards two different implied readers at the same time. ^5^ The implied reader is a streamlined version of the real reader who can be expected to understand every nuanced intention of the implied author, who in turn is responsible for “every implication, subtlety, ambiguity, and complexity that can be discovered in the text” ( 13 on page 26). The concept of the implied author is useful specifically to discern the overarching use of narrative techniques to communicate within a text ( 14 on page 10).

The political binary presented here is used as a starting point to argue for a third overlapping way to view sex/gender in the novel. Following Anne Fausto-Sterling ^9^, in her book *Sex/Gender: Biology in a Social World*, whose central tenant is “don’t get stuck trying to divide nature from nurture” (xiii), I show the particulars of the familial and sociocultural influences on Bekim as he negotiates his sex/gender. The past, symbolized in the novel as Yugoslavia generally but specifically the cultural strain originating from rural Kosovo, is filtered through Bekim in the present. The strictly differentiated and hierarchically parsed sex/gender positions of men and women in traditional Kosovan culture appear very different when viewed in the context of contemporary Finland, where Bekim’s family emigrated. Just as Robyn Warhol ( 18 on page 859) looks at the practice of ‘double reading’ to approach binaries not as opposed but as coexisting to read “texts as participating in a feminist resistance to monologism and closure,” I propose to discover how the novel at hand can shed light on both progressive and conservative readings of sex/gender and open the path for noticing the overlap of conflicting manifestations of gender that exist. In this way, we may ‘see’ gender—that often-times taken-for-granted construct—in all its generating vividity and question both progressive and conservative stances.

The most important narrative technique in the novel, which I primarily concentrate on in this paper, is the use of two narrators. ^6^ I argue that the male narrator Bekim and the female narrator Emine’s narrative sections (they’re son and mother) formally construct what can be taken as either a progressive critique of stifling normative gender roles or a conservative critique of wishy-washy gendered relativity. As in the funhouse mirror, the handling of sex/gender in each narrator’s sections can be read as a convoluted image and commentary on sex/gender in the other narrator’s sections. The key insight from the novel, regardless of political stance, is how the two narrators’ interaction illuminates our understanding of sex/gender, especially, I would argue, in its harmful outcomes for women.

*My Cat Yugoslavia*’s primary narrator is Bekim, a young man who moved with his family from Kosovo to Finland because of unrest in the Balkans. Bekim’s story tells of his unease in Finnish and Kosovan societies and cultures and outlines his coming-of-age process concerning sex/gender. The other storyline is narrated by Bekim’s mother, Emine, who tells of her youth in Yugoslavia, her marriage to Bekim’s father, Bajram, married life, the persecution of Albanians in Yugoslavia, and the family’s move to Finland, where, finally, she divorces her abusive husband and integrates into Finnish society. Bekim and Emine are both ‘autodiegetic’ (narrated by the protagonist) and ‘homodiegetic’ (told by a figure within the story) narrators of their respective sections. The novel has been termed ‘transnational literature’ ^19^ based on its themes, as well as Statovci’s life trajectory from Kosovo to Finland. The novel was published in 2014 in Finland, won the prestigious Helsingin Sanomat Literature Prize, and was adapted and staged in the Finnish National Theater. The quotes are from the English translation, which I at times compared with the Finnish original.

## Sex/gender identity as categorical and practical

Patrick Colm Hogan (2018), in his book *Sexual Identities: A Cognitive Literary Study*, differentiates between two kinds of identities. First there is ‘categorical identity,’ which is based simply on whether one fits in with the definitions and identification criteria of the category in question, including broad norms and ideals. Hogan uses the example of sex identification, which is categorical based on a “folk biological definition” ( 7 on page 12) of males and females, along with pragmatic ways to determine sex based on secondary characteristics, such as clothing, manner, hair, etc. ( 7 on page 12), hence tying sex to representation or gender. In most cases, one’s sex can be categorically determined based on evidence in an uncomplicated manner, following the norms of the socio-cultural milieu.

The rough and superficial basis of categorical identification can be compared to the more subjective reality of the lived experience of an identity, which Hogan (2018) terms ‘practical identity.’ He writes:

Practical identity is a set of routines of thought and action, including memories and aspirations, that enable one’s consistent activity in the world—particularly, the social world of interaction with others. Practical identity ranges from competence in language to one’s usual breakfast procedures to the routines that characterize one’s sexual relations, thus what Gagnon and Simon call “scripts.” ( 7 on page 13) ^7^

Here we see more variables in determining the actual lived experience of an identity, even if that identity by and large follows a ‘script’ that, through iterations, becomes routine. However, the point is that belonging to an identity and living out an identity are different. Hogan goes on to say that practical identities are “complex, not entirely consistent, varying with circumstances and mood, but defining whatever consistency we have in our thought, feeling, and behavior’ ( 7 on page 13). One’s practical identity, in all of its inconstant variation based on circumstantial mood, is often, in practice, at odds with one’s categorical identity. The crux is that, in the normal run of things, categorical identity trumps practical identity—we jettison allegiance to our practical identity in favor of upholding the validity of our categorical identity. Bekim’s practical identity, in which he has certain feminine characteristics, is an anomaly in the face of his categorical identity as a biological man.

In *My Cat Yugoslavia*, just as in the introduction above, *the* categorical identifying characteristic of the two narrators is their sex. Even if this was not a paper on the subject of sex/gender, I cannot imagine introducing these narrators without, in the very first sentence, mentioning that one is male and the other is female. The primacy of the sex category is a reflection of sex as a “socially fundamental identity,” which means it is an identity on which other identities depend ( 7 on page 12–13), such as gender, but also sexuality, occupation, position within the family and societal relations, and so on.

Applying these ideas to *My Cat Yugoslavia,* the novel can be read as critical of identity categorization, such as through Bekim’s subversion of sex/gender, which would be the case in progressive reading. In fact, the normative categorical nexus of male/masculine is a false pretense that inhibits Bekim’s practical identity, which lies between masculine and feminine norms. The angst he feels comes from falling short of the categorical norms instilled by his family, which are based on false ontological premises. From a critical perspective, we might say that Bekim’s sex/gender is present, real, and meaningful in the text, but it is not to be taken as conclusively identity-defining in the sense that it would override his practical identity. Sex/gender doesn’t have to be deterministic, as illustrated by Bekim’s lived experiences not neatly conforming to his male/masculine categorical identity.

The novel can also be read as illustrating the importance of stable categorical identities, which would be a conservative approach. Bekim’s smoldering unhappiness, unhealthy romantic and familial relationships, and isolation are a result of not finding peace with the norms of his categorical identity, which is as a man. Even if categorical norms are taken as performances or conformity, this line of thinking would argue that, for Bekim, the inability (or lack of desire) to conform to categorical norms has a destructive outcome for his place in the world and relation to it, which could be called identity. A conservative reading would posit that it is possible for Bekim to live in between norms, but since this in-betweenness leads to failure, it illustrates the desirability of conforming to the norms of his sex/gender categorical identity.

Underpinning the analysis of sex/gender in *My Cat Yugoslavia* are the inherited norms within Bekim’s family unit in relation to sociocultural contexts. Bekim’s subversions go against what he was taught in his family about sex/gender from infancy onward, propped up by elaborate customs and traditions from his culture, and embodied in the examples of his powerful and hostile father and subdued and victimized mother. This binary of sex/gender is an important aspect of the formal construction of the text, as we will see. Perhaps most importantly for now, femininity and females are defined as not-men, which is an Othering process that takes place in (or even on) the lives of females, echoing Aristotle’s line: “The female is a female by virtue of a lack of certain qualities” (qtd. in 21 on page 347). The sections narrated by Emine about her youth in Yugoslavia show what femininity entails, illuminating how Bekim’s inherited masculinity is defined. A guiding insight into the novel is that Bekim consistently grapples with the duality of sex/gender as inherited from his mother and father, but instead of rejecting femininity, which would be the definition of masculinity in a conservative context, he embraces aspects of it.

The epitaph to Part 1 of the novel, which is from Yugoslav writer Ivo Andric, supports the interpretation that Bekim consists of at least a sex/gender duality, which is expressed as masculine/feminine (leaving out animals and other potential forms of multiplicity). It is written in ‘Serbo-Croatian’ and translated as follows: “In order to see a picture of the town and understand it and its relation to the bridge clearly, it must be said that there was another bridge in the town and another river” ( 22, qtd. in 23). A single town or subject is described by the river and bridge; however, to truly know it, one must acknowledge that there is also another river or bridge. The first image is correct, but the second image is fuller. I connect this duality to two main influences on Bekim in the narrative: his mother and father. Bekim is Bajram’s son, the male offspring who “looked exactly like him [the father]” ( 23 on page 135) ^8^. However, Bekim is also his mother’s child and their narratives are intricately linked. As we will see in the remainder of the paper, these two aspects—the masculinity of his father and the femininity of his mother—vie with each other in Bekim, the subject. Overcoming the conflict from this dualism, Bekim chooses to step out of the sex/gender binary and into an alternative in-betweenness based on romantic relationships with—animals. This does not work well, as one can perhaps imagine, and so the arc of the storyline is Bekim working his way back into humanity with the broken pieces of sex/gender that he inherited from his father and mother.

## Sex/gender in two, mirroring narrators: beginnings

The beginning of *My Cat Yugoslavia* is text from a gay chatroom featuring snippets from “blackhetero-helsinki,” “OuluTop_tomorrow” and others. Bekim begins narrating as Ville, who turns out to be a “good-looking” (3) and “successful-looking” guy with a “handsome, angular face” (4) from the chat, comes to his place and they have sex, which is described in detail. Ville wants to see Bekim again, but Bekim becomes cold; he pushes Ville out of the apartment with the words “I want you to leave” (7). It’s sex, darling, not a relationship. Bekim then buys a boa constrictor at a pet shop, as described in the following section. The motivation is not clear, but there is something troubling about it, captured in phrases like “Do they [pet snakes] ever learn that all lives are not equal?” (10), and, said to the snake, “ *I own you now*” (11) and “We will be together forever […]. We would never stop loving each other” (12; italics all in original unless otherwise stated). Is the snake meant to deliver a bond that humans, despite sex but without emotional connection, fail to provide?

The binaries of male/female and masculine/feminine have been uprooted by the introduction of an animal into the romantic equation (Bekim and the snake sleep together, and the snake fondles Bekim with sexual innuendo, such as pressing itself into Bekim’s groin and giving him long, warm licks [12]) and a rejection of closeness with humans, a reoccurring theme in the novel. Comments like “I was lonely. So lonely that I sometimes spoke to myself in the apartment” (35) and “I didn’t get to know anyone and didn’t have a single friend” (32) are paired with a diatribe against his university colleagues. Bekim says, for example, “I treated them with disdain, contempt, I despised their lifestyles, their choices and problems” and “I wanted to punch them, grab them by the hair, and smash their heads against the wall or the table” (33). Coming at the beginning of the novel and bookended by romantic relationships with the snake and a cat, this can be taken as evidence that Bekim, in his ongoing struggles with sex/gender categorization, rejects humanity in favor of animals. This ‘fantastic’ element can be read as Bekim’s rejection of the delimited possibilities for sexuality and sex/gender as determined by status-quo identity politics, which is represented by the faceless masses of fellow students.

The sex/gender binary that premises Bekim’s inherited worldview comes into focus in the section called “Spring 1980: People on a Mountain” (13–23), which clearly predates the Internet era of the first section. It becomes clear that the narrative is now taking place in Kosovo and, based on heterosexual norms, that the narrator, Emine, Bekim’s mother, is speaking of herself as a 17-year-old girl who is set to be married to “the man with the beautiful smile and stubble that barely showed against the light” (13). Emine’s father, meanwhile, “was like fathers in cinema: handsome, western features and a face that narrowed toward the chin, a commanding voice and a military posture. […] He was well mannered and handsome” (13). The father’s face is “glorious and expressive” and “You could sink into it, stare at it forever” and his “hands were like two sculptures, strong and unfaltering” (14). While the father is “well respected by the locals in our village” (13), Emine’s mother “was a typical Kosovan mother and housewife. She was hardworking, good to her husband, and strict with her children” (15), which is all the introduction she gets. Females are defined as not-men, as an absence of the many qualities that define men.

In this way, the beginning of the novel introduces us to the two narrators, Bekim and Emine. What is fascinating in terms of sex/gender is that Bekim opens the narrative with a subversion of heterosexual sex/gender norms through an explicitly described sexual encounter with another man. It is not the stuff of romance, commitment, or marriage but rather pure, unadulterated and unemotional sex. In this sense, it is the stereotypical male fantasy, but with the caveat that it is performed with another male, hence capturing über-masculinity while also subverting heterosexual sex/gender norms (compare this to attachment or the reward basis for romantic love, see 7 on pages 41–42). When the narrator switches, the homoerotic register of the first section appears to overlap with Emine’s telling: men’s physicality, handsomeness, and masculinity are emphasized. Upon a quick first reading, one may not notice that the narrator, or at least the narrator’s sex, has changed. The descriptions of men’s physicality are similar in both sections. Yet, this is a 17-year-old girl talking, for the most part, of her father. Males and masculinity are desirable, respected, and praised, while womenfolk are talked about in terms of being “hardworking” and in relation to their husbands, to whom they must be good wives and mothers. 

Following Catherine Romagnolo ( 24; 25 on pages 43–56), the beginning of anything, but specifically a novel, is crucial: “beginnings invoke the power of authority, authorship, and tradition” ( 25 on page 44). Beginnings call into being the subject positions of the reader vis-à-vis the writer and produce what Susan Lanser calls “discursive authority,” which is the positioning of the text’s various levels, including narrator(s), as having “intellectual credibility, ideological validity, and aesthetic value” ( 26 on page 6, qtd in Romagnolo 25 on page 44). In other words, Statovci hasn’t begun the novel with internet-age hook-up sex just for the fun of it ^9^. Statovci aims to evoke queerness, homosexual sex, and the narrator’s positioning as a queer man, and hence his break with normative sex/gender categorization. Everything that follows, including Emine’s strict adherence to her sex/gender category, can be read through this initial value claim.

Consider this description of Bajram on Emine’s wedding night: his
proudly engorged penis looked frighteningly large. His arms were muscular and the contours of his body were well defined, his shoulders broad; everything about him was symmetrical. He had a handsome washboard stomach and a back that curved into buttocks so round that they appear almost separated from his body. The lower part of his back and his coccyx was covered in a thin layer of hair that seemed ready to thicken and conquer a larger area for itself at any moment. ( 23 on page 122)

This is certainly an erotic description that, coming from Emine, is heterosexual; however, when read through the beginning of the novel and its homosexual premise, it can also be taken as homoeroticism based on the initial ‘discursive authority’ of a queer positioning. While Bekim mimics Emine’s feminine position in romantic relationships, as will be shown, Emine also mimics Bekim’s homoerotic register, which is only mimicry because it is read after the novel’s opening gay sex scene, and if we do not hold homosexuality as an aberration from heterosexuality. In a gay novel, the positionality that *My Cat Yugoslavia* is claiming in its opening, heterosexuality is the aberration. In this case, it can be said that Emine borrowed Bekim’s homoerotic language to describe Bajram.

Via the two narrators, the structure of the novel can be taken not only as polyphony and representing heteroglossia, but also—by the narrator’s discourse being read as paired with the other narrator—as dialogic and double-voiced discourse. This means that whatever meanings we discern here is dynamic and based on the interaction of the two narrators’ sections. The subjectivity of the narrator is taken together with the other narrator to whom s/he is paired. The differences can be parsed into ontological, contextual, linguistic, ideological, and idiolectical dialogisms, which means that the subjectivity of the speaker is taken together with the context of the uttered language—for both narrators simultaneously ^27^. This diversity of voices, which is heteroglossia, is a defining characteristic of the novel according to Bakhtin ^29^. *My Cat Yugoslavia* uses this narrative technique (albeit at a more structural level through two narrators’ separate sections) to highlight the outcomes of different kinds of identity categories concerning sex/gender. Bekim and Emine are both, for the bulk of the novel, miserable in their sex/gender categories but for different reasons. As a woman, it is clear why this is the case for Emine: Being a woman in the traditional Kosovan context, which establishes the norms of sex/gender in the novel, is simply a bad deal. Being a man is hugely advantageous. The fact that Bekim rejects this advantage (at least partially) may be due to solidarity with his mother, whom he protects from his abusive father. Bekim spends a significant portion of the narrative mimicking his mother’s gendered positionality, which I will show next. This will correlate back to the question of conservative and progressive readings of the text.

## Stable sex/gender categories and their inversion

From her youth in Yugoslavia and throughout the bulk of the novel while married to Bajram, Emine’s reality is overwhelmingly shaped by her sex and correlating gendered roles. While still a child, Emine was allowed to accompany her father to the market in town to buy provisions. One day, however, a man treats Emine as a sexual object and her childhood, and hence her relative freedom from the constraints of sex/gender, is over. Emine’s father blames her for the man’s behavior and never takes her to the market again. The constriction of female movement, public appearance, and many other aspects are unquestioned because they are rooted in the body, exemplified here in Emine’s breasts, which have just begun to appear.

Childhood is also relatively pre-sexed and gendered because Emine is still illusioned about her future and prospects. She imagines that she will become a famous singer, like the ones she sees on TV. As she grows, she realizes that she is not much of a singer, she’s an average student, and is not talented at anything; the only thing she will be good for is being a wife. This initiation into the constricted realities of womanhood soon gives way to a suitor, Bajram, who then becomes her husband. Wedding traditions are described in great detail, almost anthropologically, as the text illustrates the cultural mechanisms by which gender is installed on the bodies of men and, much more so, women. The minutiae of traditions, which, we are told, differ from village to village, are followed solemnly: the social standing of the family depends on performing these traditions correctly ^10^. Hence, society enforces the upholding of these gendered markers, which, in the case of Emine, is in practice orchestrated by her mother. This is a case of the subjugated sex upholding and continuing the mechanisms of oppression (gender).

The traditions themselves are mostly quirks that seem harmless in and of themselves, but they work to naturalize the position of women as subservient in the familial and social hierarchy. For example, when, before her wedding, Emine must bid farewell to her female family members, neighbors, and friends, she must perform an inconsolable sadness at losing her home and loved ones—for she will now ‘belong’ to the family of her husband. Physically, she’ll move to his house. If she can’t cry, “ *You’ll just have to pretend. Lie so much that you can feel it throughout your body and soul, right down to your fingertips*” (72), the mother instructs. The role of affect in underpinning the severity of this cut acts on Emine psychologically: it frames this reality as absolute and unquestioned. Emine’s mother also advises her to hide a razor in order to make a small cut in her armpit, from which she can spread ample blood on the marital sheets to avoid any unpleasant speculation. Marriage is a transaction—also supported by the great material wealth in presents that the groom presents to the bride’s family—with the woman, as a producer of children and household servant, as the untarnished object that is bought.

Emine’s role as a woman is made clear through her transition from child to female adult, the wedding rituals that symbolically cement her position as an owned object, and the example of her mother and other women in the village. Each of these is represented as a doubled non-male positioning, which conversely defines everything that male masculinity *isn’t*. As such, Emine’s descriptions define, in the sociocultural and familial context, what Bekim should be as a man. It appears that, since women are getting such a bad deal, there are many more social interventions in maintaining women’s gendered roles. For men, traditions and rituals, as represented in the novel, are minimal. In fact, one of the traditions is that the groom’s male relatives fetch the bride to the wedding, but Bajram ignores this tradition and arrives himself. Still not married, but riding in a car together alone, Bajram first puts Emine’s hand on his crotch and then, when she demurs, slaps her across the face. This event tells you everything you need to know about Bajram and Emine’s marriage and highlights the differences between the genders: Emine’s every movement, expression, and behavior is orchestrated and choreographed based on the rules of tradition as dictated by the norms of society, but Bajram, because he is a man, is uninhibited in his actions, speech, and behavior. Emine is slapped for attempting to dictate how she is touched, which is expressing agency, while Bajram does exactly as he pleases.

In terms of Emine and Bajram, the conservative and progressive readings will be quite similar. The radical differences between sexes, based on essentialism, are taken too far to admit much space for discussion. The political readings begin to pan, however, when Bekim takes on similar feminine qualities as his mother. As a narrative technique, this is the transposition of elements from one narrator into the section of another narrator with very different contexts (Bekim’s sections take place in Finland, and he is, of course, a male). Following David Herman (1998), this can also be thought of as polychromatism in which the gendered characteristics are rooted in “more than one place at a time” ( 30 on page 75), which extends from Bekim backwards to Emine, her mother, and so on. However, because each narrator has a distinct temporality, this is not strictly the polychronic narration that Herman discusses, which he defines as “narration that exploits indefiniteness to pluralize and delinearize itself” ( 30 on page 75), but rather an overarching design that can be attributed to the implied author ^16^ that, in terms of creating meaning through form, has much in common with polychronic narration. The feminine role is carried out similarly across multiple generations in the narrative, and, in this way, the Kosovan gender lives on in Finland in the 21 ^st^ century. Because the two narrators’ sections are combined to create one narrative, femininity ‘happens’ across those generations in close proximity within the text. The novel’s trick, of course, is that Bekim is a man, hence the schemata is thrown on its head by switching the sex of the subject in question. 

This technique suggests that the text is commenting on the characters’ relationship as a mother and son. More importantly, the narrative goes beyond progressive and conservative takes on sex/gender to show how strongly normative associations of sex/gender can be rooted in cultures and readings. If one set of gendered codes is familiar when referring to women, why might the same codes, when performed by a man, come across as more or less obscene? This not only illustrates the unseen quality and force of normative sex and gender alignment, but also might, depending on our reactions to it, illuminate our political beliefs concerning sex/gender and allow us to ‘see’ gender afresh.

Bekim’s process of inhabiting an in-between sex/gender role can be understood through his association with his mother against his father, Bajram. To begin, Bekim must reject (or ‘kill off’) his father, as in the Oedipus complex. In Freudian theory, the oedipal stage is when the individual first becomes aware of sexual characteristics and differences in the other sex, and usually focuses attention and affection on only one of their parents, which has been seen as important for the individual’s later gender identity and self-concept ( 31; see also 32; 33). Although criticized in other schools of psychology, the Oedipus complex retrains its cultural reference to parents, children, and gender. Bekim says,

For the majority of my childhood, I’d wished my father would die. I prayed. Whenever I saw him thrust his empty plate in front of my mother’s face without saying a word—she was supposed to realize this meant he wanted more food—or whenever he threw his stinking socks in her lap […] I knew he would never bring happiness to anyone. (90)

Bekim’s wish for his father’s death is linked to Bajram’s treatment of Emine and is based on normative gender roles: the man must be served without him having to utter a word; traditionally, the woman’s worth is tied to how well she performs this service. The silent plate-thrusting that enrages Bekim is also told later in the novel from Emine’s perspective. The family has male majority-culture Finnish ^11^ visitors when Bajram performs this humiliation tactic; the visitors lower their eyes in pity and shame, while Emine feels helpless. Faced with such injustice, it is possible that Bekim not only rejects the masculinity of his father but also embodies the femininity of his mother in solidarity against the father.

The main way in which Bekim exhibits femininity is through a relationship with a humanoid cat whom Bekim meets at a gay bar and falls for romantically. The cat is a jerk: he’s homophobic, hates immigrants, and, for example, upon meeting Bekim, tells him “a *frightful name* […] *utterly dreadful, ha ha* […]. *Bekim. It’s such a dreadful name I’m sure I never want to hear it again!*” (54). Despite saying such things, which are linked to Bekim’s position as an immigrant Finn, Bekim invites the cat to live with him, much as Emine goes to live with Bajram after being slapped in the face.

Bekim’s relationship with the cat is dysfunctional in much the same way as Emine’s relationship with Bajram. There are many parallels, and Bekim is coded as having a feminine role when compared to Emine and Bajram’s relationship. For example, Bekim’s apartment’s “walls, floors, windows, and tiles were all sparkling, not a speck of dust in sight” (page 76), which echoes Emine’s description of a proper Kosovan house, which is a “reflection of the hostess.” According to Emine’s mother, “a Kosovan home should always look tidy and shouldn’t look lived in. […] [A] tidy home made a woman whole and made a girl a woman” (48). The cleanliness of the home in Emine’s telling is decidedly gendered as a feminine attribute and responsibility—it makes “a woman whole” and a “girl a woman.” Bekim’s compulsion to clean, a reoccurring theme in the novel, can hence be coded as feminine from the standpoint of Kosovan gender norms.

This kind of subservience through cleanliness is framed as a way of showing devotion or being a good ‘wife’ despite the man (or cat) behaving detestably. The cat says to Bekim, “ *Aren’t you fat and lazy, lounging around like a slab of meat!*” (78) and “You’re ugly. Butt ugly, if I may say so. That’s a bad expression for you. Change it *this instant*” (80, italics added). Note also that elsewhere Bajram wants Emine to answer “ *this instant*” (110), hence repeating the same phrase to reflect the cat and Bajram’s similarities. Nevertheless, Bekim says, “I wanted to be good to the cat, to meet his needs and requirements” (76). He goes on to say, “I’d taken it upon myself to put the cat’s clothes in the washing machine, I’d ironed his shirt, polished his shoes, and aired his woolen coat. That’s how much I liked him already” (80). Compare this with Emine, who says, the same day she is slapped and sexually attacked in the car, “ *I will be the perfect woman for him*” and thinks of the tasks she’ll perform “impeccably”: “ *Everything he wants, his wife shall give him*” (112). She goes on to say, “I was the wife of his dreams. I washed, clothed, fed, and looked after his children, scrubbed and polished his guests’ shoes when they visited the house. […] Every evening I laid out his clothes for the next day, I ironed his socks and shirts and washed his underwear in almost boiling water” (135). The text is incongruous in Bekim and Emine’s sections in precisely the same way: despite being abused by their partners, they clasp onto the motivation to be perfect providers and partners—they both iron shirts and polish shoes. The only difference is that Bekim is a man. It is traditionally understood that women must suffer at the hands of men, captured even in geography: Bajram is from a region “renowned for its traditional ways, its violent men and unhappy women” (71). Tradition dictates that men are violent and women are unhappy, so Emine’s situation is normalized. Emine may not be able to leave her husband, but Bekim, as a man, should be able to kick the bum cat out on its tail, something he never does (the cat ends up leaving him). Bekim’s inability to do so is a reflection of him taking on a feminized gender position.

The correlations, unfortunately, continue: “nothing pleased him [Bajram], and he flew into a rage with increasing regularity” (137) just as the cat, who is slovenly, lazy, and has become “fat and bitter” (106), expects Bekim attend to all his whims. The cat then screams, “ *you’re so pathetic*” and “I never thought I hated all immigrants, but apparently I was wrong. And I never thought I hated you, but it turns out I hate you most of all” (115) and attempts to kill him in a manner reminiscent of how Bajram comes close to killing Bekim when Bekim defies him. The last words the cat says to Bekim in the novel are “ *Nobody will ever love you. […] Nobody. […] You’re going to die lonely, alone and depressed [….] And you deserve every bit of it. Wog*” (119–20). 

In addition to the racist term ‘wog,’ ^12^ this is a classic example of shaming, which is belittling the inherent worth of another. Bekim is, according to the cat, essentially an un-loveable creature. Shame, or rather the lack of shame, is important in Kosovan culture. Emine links it to cleanliness when she says, “An Albanian is prepared to die to preserve his honor and keep himself from losing face, because losing face was a fate many times worse than death. […] Albanians refused to feel any form of shame” (48; see also 10 on page 79; 34; 35). As such, Bekim’s father is prepared to disown his child Bekim for defying him. Rather than feel shame, the culprit who has brought shame of the family is driven out and the shame is absolved. However, Bekim is a Finnish child, brought up in Finland. He says, “I learned to speak and read in a language he [Bajram] didn’t understand, to live among people whose culture he despised, to talk about subjects he couldn’t fathom” (87). And emotionally, Bekim is also Finnish (difference can lead to shame; see 36 on page 46 for ‘equality as sameness’ in the Nordic context, also ^37^ for Finland) and says, “As a child the first emotion I fully recognized was the feeling of shame. When my parents asked for driving directions in mangled Finnish I sank into the backseat and tried to make myself so small that nobody would see me and felt utterly ashamed” (91). If a lack of shame is Albanian, feeling shame is Finnish, and Bekim has not only symbolically drifted from his father, but in practice has joined another culture, which in the context of the text’s depiction of Bajram— that is, his hatred of Finland and Finns—is among the worst things Bekim could have done. Because the Finnish welfare state is a boon to Emine as a woman, in the shape of daycare and schools for children, so that she has less of a burden, I take Bekim’s Finnishness as his alignment with his mother against the father. In terms of the novel’s portrayal, Emine’s ability to leave Bajram is only conceivable in the Finnish context of safe houses and government-provided housing. By becoming Finnish, Bekim is moving away from categorical male masculinity, as defined in Kosovan culture in the novel.

In a conservative reading, the incongruousness of Bekim’s situation is based on the fact that he does not embody sex/gender norms. By giving up the stable positioning as a masculine man, he is made to suffer as a woman. If he could embody his normative sex/gender role, he could be liberated from such suffering by, in the first place, not taking such abuse from the cat. This would not answer to Emine’s suffering in the novel but would at least liberate Bekim from following in her footsteps. A conservative reading would also define Bajram as an aberration of masculinity. Instead of abusing one’s power and harming one’s family, ‘healthy’ masculinity would provide care, much as Bekim later provides for a street cat to which he speaks to in a “fatherly” (157) tone, bathes, feeds, and saves. Compare this to some traditional Kosovan weddings in which the villagers snare a street cat and lock it up. The groom is to bring the cat “to his newly wed bride on their wedding night and kill it with his bare hands to demonstrate to his wife his supremacy, to teach her to fear him” (28). Emine is told this story by her mother before her wedding, and cannot help but think of “the cat’s neck snapping,” the blood and smell. Both represent versions of masculinity, albeit polar opposites of one another. One need not endorse a drastic form of masculinity to uphold the conservative premise outlined here, which could align with the ‘fatherly tone’ variant of masculinity.

A progressive reading would hold that Bekim’s in-between sex/gender positioning may in fact be a stage in a progression towards being liberated from the hostile masculinity of his father, which is the source of much of the suffering in the narrative. On a more symbolic level, Bekim is disowning his membership to the patriarchy and siding with the victims of gendered inequality. This may be to his own detriment, but at least he is not adding more suffering by continuing the traditions of hostile masculinity that are his birthright.

## Bajram as the begetter of trauma

Bekim’s hatred of his father, Bajram, has been shown above. No matter how the text is interpreted, there is no getting around Bajram’s centrality to Bekim’s troubles; Bajram is referenced throughout the text, and entire sections are dedicated to him. Bajram continues to be central to his son’s musings even after the father has moved back to Kosovo and Bekim never sees him again. He remembers his “father’s absence” often: sometimes it makes him “so angry I wonder how it’s possible for someone to carry this much anger inside” (44) while other times it “doesn’t feel bad at all, like a bowling ball resting on my stomach” (44–5) but at night he’s “so bitter that [his] whole body feels about to burst into flame” (45). Sometimes, Bekim misses him, “his voice,” and when he can barely remember his father, he pulls out photos that he does not look at and then puts away and rather goes for a run, cleans, or reads (45). Bajram joins these activities, however, pushing “his way in between the lines” while reading, jogging besides him, or “when I wash up the same glass so long that it cracks, he is there in the cut on my hand spitting blood down the drain” (45). Simplest of all, the absence comes as tears.

Based on the reoccurrence of Bajram interjecting himself into Bekim’s thoughts and memories, as well as the abuse Bekim suffered, one could conclude that Bekim suffers from trauma. This is evident from Bekim’s description of how Bajram’s image, described as his absence but present as a memory or even a figure that jogs beside him, pushes itself into Bekim’s consciousness, even when he tries to avoid Bajram. According to Cathy Caruth ( 38 on page 4), “trauma is not locatable in the simple violent or original event in an individual’s past, but rather in the way it’s very unassimilated nature—the way it was precisely not known in the first instance—returns to haunt the survivor later on.” Mario Grill ( 39 on page 59) links this form of ‘haunting’ and trauma to polychronic narration, which showcases “the unassimilated nature of […] trauma and how it is not locatable in the original event.” Likewise, Bajram’s behavior is temporally delinked from the moment of narration and the described moments in which he pushes his “way in between the lines” (45). The trauma inflicted by Bajram is present in the past, present, and, presumably, future, hence qualifying it as a polychronic narration.

The last images of the novel include Bekim contemplating Bajram’s suicide: “Was he thinking about me, was my father thinking about me at the moment he finally refused to carry on living, in such violent fashion?” (255). Here, the dread and violence of the memory is tinged with compassion, as when Bekim says “he finally refused to carry on living,” implying that life was too much, too difficult for Bajram. Bekim continues, saying, “I never got an answer, but I’m sure that’s what my father was thinking” (255). Given the centrality of Bajram to Bekim’s psyche along with his reoccurrence, this statement is pregnant with meaning. But what does it mean? Ambiguity reigns.

Initially, and given the tone of the section, the phrase could mean that Bajram is thinking of Bekim in terms of affection, perhaps asking for an apology, and making peace. Earlier in the text but at a late stage of the fabula Bekim imagines that his father would introduce him to his friends proudly, “like a trophy” (23). This seems imaginary given Bajram’s personality and their relationship; Bajram has violently disowned Bekim. Therefore, Bajram might also think of Bekim in terms of desperation, hate, and vengeance, especially as Bajram’s next action is to shoot himself, an incredibly violent act. Bekim also mentions that when Bajram talked and fantasized about death, which he did often, “I wasn’t sure whether he really wanted to die or whether all he wanted from death was what it would mean for his loved ones” (253). This implies that Bajram might be thinking about how Bekim will suffer from his death. However, Bekim is relieved that “he [Bajram] had finally found a way to turn to the only option still open to him” (252). In Bekim’s view, Bajram’s life was a failure. At the heart of the failed life is Bajram’s form of masculinity, which the novel charts from the early days in Yugoslavia through living in Finland. Bajram’s life is defined by violence, that famously masculine arena, from his first time slapping Emine before their marriage to his ultimate murder—of himself. However, despite the evidence of Bajram’s belligerence and malice, Bekim, upon meeting his mother, only asks about is father. “[H]e didn’t ask anything about me,” Emine says, “he asked only about his father though I was still here and his father was not” (232). Despite everything, Bekim is drawn to his father, and so, perhaps, he imagines that his father is also secretly drawn to him.

Adding to the ambiguity, and hence leaving readers’ interpretative window open wide, Bekim is an unreliable character and narrator. His unreliability with romantic partners begins when he tells the cat about his life. Bekim says, “I was tailoring my story to what I thought he wanted to hear” (64). As such, he invents the backgrounds of his parents and siblings: his parents were medical doctors but died, his siblings are academics. “ *[W]e’re not like other immigrants*” (64), he tells the cat, who is anti-immigrant. The cat is impressed by Bekim’s “genes,” which he mentions a few times, perhaps linking his anti-immigration stance to eugenics. None of what Bekim tells the cat about his family is true.

Later, when Sami presses Bekim about his father, who has died at this point, Bekim tells a version of the story that is incompatible with what we know of Bajram, which is quite a lot. Bajram, who once robbed a supermarket with a friend when he was unemployed and needed money (203–6) and kicked his daughter because Emine suggested that the kids needed another bedroom (164), hides, according to Bekim’s telling to Sami, under blankets and says, “ *Sorry, Daddy’s very scared right now*” (254). This tone is impossible to imagine coming from the Bajram readers know thus far—the word ‘Daddy’ is inconceivable in Bajram’s mouth. However, in the Finnish original it is not ‘Daddy’ but the neutral *isä*, as in “anteeksi, isää pelottaa todella paljon nyt” ( 40 on page 283). Perhaps the translator, David Hackston, consulted Statovci over this tonal question or based the translation on the out-of-character (for Bajram) phrase “ *very scared right now*,” which is a direct translation. However, this is not the Bajram readers have come to know.

Further, Bekim says he “stroked his [Bajram’s] damp back” and mopped up vomit on the floor next to the bed, although Bekim hated him. To this story Sami says “Thank you” (254), which could indicate ‘thank you for being vulnerable.’ However, the one giving thanks, the one who received something, was Sami. He was told just the kind of story that would help make sense of Bekim, his father, and the latter’s death. That the story is not true, or at least contradicts everything that has been said about Bajram and Bekim’s relationship, seems to indicate that Bekim changed his identity to suit the lover in question.

This changeability, based on lies, is a weak position from which to develop relationships with other humans. Bekim’s unreliability illustrates, for the conservative reading, what relativism can lead to: without stability concerning the ‘facts’ of our lives, how can we begin to really know another or ourselves? This extends to ignoring the categorical definitions and norms of sex/gender, which, according to the argument, can lead to confusion at best and chaos at worst. Although Bekim appears relatively happy at the end of the novel, the implication is that a relationship built on disbalanced agency and power dynamics and lies is doomed to failure, just as Bajram and Emine’s relationship failed.

Bekim’s inconsistencies and lies remain ambiguous and obscured in the narrative and make him an unreliable narrator. Such unreliability is a tactic that Bekim learned from his father, which illustrates the similarities between father and son. Bekim tells his father how he was harassed by kids for being an immigrant and poor—they even tore the sleeve off his jacket. Bajram advises, “ *Don’t ever tell them your name, and don’t say where you come from.* […] *Don’t ever tell them who your parents are, who your siblings are, don’t get in people’s way, and don’t talk, and if they come and ask you something, you know what to do.*” Bekim knows the answer: “I’ll lie.” Bajram answers, “That’s right. You lie” (246). Bajram’s advice to his son for being an immigrant in a foreign land is to emasculate himself and, if that does not work, to lie. Bajram tells majority-culture Finns that he is Belarusian, Russian, and anything but the truth (211). It may not be a coincidence that Bajram advises Bekim to not tell others about his parents and siblings, which is precisely what Bekim does with the cat: Bekim tells the cat fabricated information about his parents and siblings. The cat tries to locate these fabricated siblings, and once he cannot, he flies into a rage that helps lead to their break up.

At the end of the novel, Bekim appears happy with Sami and thinks, for example, “Did my father ever experience anything like this?” (254), which means holding hands and feeling the breath of a lover. This can be taken straight-forwardly. Bekim has overcome great adversity and trauma to lead what appears to be a rather contented life. His identity is neither the hostile masculinity of his father nor the victimized femininity of his mother, but rather something in between. This would be the progressive reading. However, the degree to which he has found happiness with Sami at the end of the novel is suspect, since it is built on false pretenses that will eventually unravel, says the conservative interlocutor. Bekim remains isolated even from the person closest to him, while he continues to lack agency in the relationship—cleaning and remaining for days at home, which Sami at one point calls “pathetic” (228), while Sami goes out into the world to work as a bank manager. Again, such a reading might overlook the fact that if Bekim were female, such an arrangement would not stand out as much.

Bekim’s wording in this section is also alarming. He says that Sami is wearing “clothing I have washed for him” (254), which may link to the dependence that Bekim enjoys in owning a snake (30). After a temporary breakup, Sami returns to Bekim’s apartment, and Bekim cannot sleep.

Once I’d ironed and folded all his clothes I fetched the shoe brush from the hallway. I brushed the loose mud from his shoes and took them with me as I slipped outside, sprayed them with protective lacquer, and when I came back inside I polished them and watched them dry until I placed them on the shoe rack and went back to bed, lay next to him, and fell asleep in an instant. (247)

While Bekim is performing these tasks, he’s thinking about his father but he is *performing* his mother. Bekim is comfortable in feminine roles, as defined in his family. This quote closely resembles Emine’s quote, in which she cares for guests’ shoes. This service gives women worth in Emine’s culture. Here it appears to do the same for Bekim and, once it is done, he can sleep “in an instant,” which indicates peace. While Bekim fulfills a feminine role, Sami is stereotypically masculine—messy, unaware, leaving behind a dirty frying pan, etc. In the English translation, the masculine pronoun ‘he,’ referring to Sami, appears 14 times in a single paragraph (227). However, Finnish has gender-neutral pronouns ( *hän*), so the language is not gendered in the original as it is in English. However, the age-old marital stereotype of the messy and ignorant man leaving dirty laundry on the floor and the martyred long-suffering woman who “scrubbed” and “scoured” the bathroom tiles (227) is abundantly clear also in the Finnish. For example, Sami returns to Bekim’s spotless apartment and does not notice that his shoes are muddy. He leaves them and other things lying around. Bekim performs his caregiver role ‘ *impeccably*’ (to borrow Emine’s word), even watching the polish dry. Sami will be off to his well-paid job in the morning, and Bekim will wait for him in the apartment, probably cleaning the already spick and span apartment.

## Conclusion

In this paper, I created a heuristic of progressive and conservative readings of sex/gender in the novel based on male and female sexes and their corresponding gender constructs. This allows us to map out a spectrum of possibilities concerning sex/gender, or specifically, how it manifests in various ways to different kinds of readers. Accepting that various ideas concerning sex/gender exist and that they can be supported by evidence in the text allows for the complication of the binaries in what Robyn Warhol ( 17 on page 858) would call ‘doubleness.’ Rather than understanding binaries as opposed, they can be viewed as coexisting. Warhol writes, “To be ‘double’ is to resist categorization as one thing or the other; to invoke ‘doubleness’ is to address binary oppositions without resting comfortably in either of the two terms being opposed” ( 17 on page 857). This expands our understanding of binaries by admitting an array of possibilities between two poles. 

As such, the most important insight from the progressive and conservative readings outlined in this paper is that they shed light, through the switching of normative sex/gender positions, on the nefarious invisibility of what largely passes as feminine gendered roles. A woman polishing shoes and watching them dry—as Emine does—may go unquestioned within the power dynamics of gender in our society, readings, minds, and lives. Bekim’s performance of ‘femininity’ makes gender visible through this narrative technique. Progressive readings concerning Bekim may be forced to reassess what his subservience in relationships entails from a sex or gender perspective. Conservatives may pause to question the assumption that gendered roles are based on a harmonious link with one’s sex. In my reading, the novel forcefully illustrates that gender is sometimes just a pretext for the abuse of power. The novel artfully illustrates what it is to be a victim of gendered relational dynamics and power—even if in this limited capacity that is open to a multiplicity of different readings beyond those presented here. Similarly, Bajram’s form of masculinity leads to failure. His family has been torn apart and traumatized, and he commits suicide alone. Sex/gender, as traditionally defined for Bekim via his family, brings no good to anyone.

Therefore, instead of taking gender as a binary based on the dimorphism of sex categories, it would be better to approach it via doubleness, as referenced by Warhol above, or as a spectrum of mulitple possibilities beyond such a delimiting dichotomy. Femininity and masculinity need not be oppositional, but can coexist in one subject, as illustrated by Bekim, and lead to a positionality beyond doubleness. However, it can be argued that Bekim does not succeed in taking what is best from masculinity and femininity but rather is (in part) a product of reactive forces that he sorts out the best he can. The goal was to find the middle ground between the progressive and conservative readings, which, based on such a premise, must compromise by admitting that binaries are the way many people understand sex/gender. I conclude that, despite binaries being a prevalent method of thinking about sex/gender, we may also look into the nuances in which doubleness and overlaps occur. One aspect need not exclude the other but can rather lead to a multiplicity of possibility. Sex/gender is never a valid excuse for what is actually the abuse of power. Bekim and Emine’s twin sections make this abundantly clear.

At the formal level, Statovci’s novel invites a wide array of interpretations due to its ambiguity. Most importantly, the two narrators allow for a dialogic discourse that opens vast vistas of possibilities across sex/gender, time, place, affect, laws, and familiar, cultural, and societal norms. The narrative gap left open by the ambiguousness of this dialogic narration will be filled in and interpreted by readers, which means that the final political message resides within them. However, by showing gender across time-place for male and female characters, the dialogic narration makes gender more visible, which is an important step in questioning inherited ‘folk’ understandings of sex/gender.

## Ethics and consent

Ethical approval and consent were not required.

## Data Availability

No data associated with this article.
