# Supplementary material for: Sex/gender conservatively, progressively, and in-between: The politics of ambiguity in Pajtim Statovci’s My Cat Yugoslavia
Source: Open Res Eur. 2026 Jan 18;5:235. Originally published 2025 Aug 8. [Version 2] doi: 10.12688/openreseurope.20789.2 (PMC12831046; doi:10.12688/openreseurope.20789.2)
Supplement: Peer review history

# Reviewer report: Reviewer response for version 2

**DOI:** 10.21956/openreseurope.24698.r68343

**Published:** 2026-02-05

**Authors:** Singgih Daru Kuncara

**Affiliations:** 1 Universitas Mulawarman, Samarinda, Indonesia

This manuscript presents a fresh perspective on the "politics of ambiguity" in Pajtim Statovci's *My Cat Yugoslavia*. It effectively argues that textual ambiguity serves as a political tool, allowing for diverse and valid readings of sex and gender. Through the concept of "structural dialogism," the author demonstrates the originality of this argument. The alternating narratives between Bekim and his mother, Emine, disrupt traditional gender roles and invite readers to fill "narrative gaps" based on their own beliefs.

 The article successfully integrates secondary sources and relevant theoretical frameworks—such as Fausto-Sterling's sex/gender nexus, Hogan's categorical and practical identities, and Warhol's concept of "doubleness"—to deconstruct binary normative assumptions. Consequently, this article makes a significant contribution to queer studies, gender theory, and transnational literature. It achieves this by centering the position of women, particularly Emine, in the face of male violence as a primary political issue rather than mere background to Bekim's story.

 However, the presentation of the manuscript is rated as only "partially" effective in terms of clarity and cogency due to a theoretical density that may overwhelm the reader. Frequently, the author introduces complex concepts simultaneously—such as polychronic narration, the implied author, and Bakhtinian double-voicing—without allowing sufficient space for each structure to develop. Additionally, the latter sections contain excessive plot summaries that disrupt the analytical flow. These summaries should be condensed to better emphasize the author's interpretive claims. The author is advised to focus on a select few frameworks most critical to their argument and ensure that every piece of textual evidence is directly linked to a discussion of political significance or narrative form.

 The persuasiveness of the argument is also rated as "partially" successful. The "conservative vs. progressive" heuristic employed is too rigid and simplistic, risking the reduction of the novel's complex ambivalence into a binary debate. Furthermore, the use of judgmental terms such as "healthy identity," "abnormal," or "wishy-washy gendered relativity" is problematic. While intended to describe "folk psychology" perspectives, these terms may unintentionally reinforce normative ideas or pathologize characters. To enhance the scientific soundness of the research, the author must revise this language—for example, by replacing these terms with "normatively legible identity." Finally, the author should expand the gender analysis to be more inclusive of race and migration, and include a methodological paragraph discussing the geopolitical limitations of the "conservative" and "progressive" labels to avoid generalization.

If any, are all the source data and materials underlying the results available?

No source data required

Is the work original in terms of material and argument?

Yes

Is the work clearly and cogently presented?

Partly

Is the argument persuasive and supported by evidence?

Partly

Does the research article contribute to the cultural, historical, social understanding of the field?

Yes

Does it sufficiently engage with relevant methodologies and secondary literature on the topic?

Yes

Reviewer Expertise:

Literary Criticism, Literary translation studies

I confirm that I have read this submission and believe that I have an appropriate level of expertise to confirm that it is of an acceptable scientific standard, however I have significant reservations, as outlined above.

# Reviewer report: Reviewer response for version 2

**DOI:** 10.21956/openreseurope.24698.r68268

**Published:** 2026-01-23

**Authors:** Ralph J. Poole

**Affiliations:** 1 University of Salzburg, Salzburg, Austria

The revised version of the article has substantially addressed the reviewer's suggestions. It is now an even more comprehensible and significant contribution to queer literary scholarship.

If any, are all the source data and materials underlying the results available?

Yes

Is the work original in terms of material and argument?

Yes

Is the work clearly and cogently presented?

Yes

Is the argument persuasive and supported by evidence?

Yes

Does the research article contribute to the cultural, historical, social understanding of the field?

Yes

Does it sufficiently engage with relevant methodologies and secondary literature on the topic?

Yes

Reviewer Expertise:

gender studies, queer theory, literary studies

I confirm that I have read this submission and believe that I have an appropriate level of expertise to confirm that it is of an acceptable scientific standard.

# Reviewer report: Reviewer response for version 2

**DOI:** 10.21956/openreseurope.24698.r68347

**Published:** 2026-01-23

**Authors:** Alan Ali Saeed

**Affiliations:** 1 University of Sulaimani, Sulaymaniyah, Kurdistan Region, Iraq

This article offers an ambitious and intellectually engaging reading of Pajtim Statovci’s *My Cat Yugoslavia* through the lens of textual ambiguity and its political consequences for interpreting sex/gender. The author’s central argument, that ambiguity enables both conservative and progressive readings of sex/gender, while also inviting overlapping interpretations that resist closure, is both timely and productive. The essay is strongest when it links narrative form to ideological uncertainty, especially through the alternating narrators, Bekim and Emine, and when it frames this alternation as a structural dialogism that destabilises each voice. The manuscript demonstrates wide theoretical awareness, drawing on Fausto-Sterling, Hogan, Todorov, Iser, Caracciolo, Guédon, Bakhtin, Warhol, and trauma studies, among others. This breadth of reference gives the piece an impressive scaffolding, and it will likely appeal to scholars working in queer studies, gender theory, narratology, and post-Yugoslav/transnational literature.

 At the same time, the article occasionally risks overextending its interpretive reach by leaning heavily on the progressive/conservative binary as a guiding heuristic. While the author explicitly acknowledges the simplification, the framework sometimes produces a slightly schematic movement in the argument, where the complexity of Statovci’s novel, particularly its racial politics, migration anxieties, and surreal animal eroticism, threatens to exceed the analytic container. In addition, the manuscript would benefit from some tightening of structure, greater consistency in terminology, and more careful handling of evaluative language (for example, “healthy identity,” “wishy-washy gendered relativity”), which may inadvertently reproduce normative assumptions even when deployed descriptively. These are not fatal weaknesses; rather, they are opportunities to refine an already promising contribution.

 One of the most compelling achievements of this paper is its insistence that ambiguity is not merely an aesthetic flourish but a political engine. By treating ambiguity as a “narrative gap” in the Iserian sense, the author convincingly argues that readers do interpretive labour that is shaped by their own ideological positions. This is an insightful way to explain why *My Cat Yugoslavia* continues to generate disagreement across interpretive communities. The emphasis on “hesitation” also resonates well with Todorov’s theorisation of the fantastic, and the manuscript usefully extends this hesitation beyond the supernatural into the domain of sex/gender, identity, and social legibility.

 The author’s close attention to narrative structure, particularly the alternating sections narrated by Bekim and Emine, is another clear strength. The reading of these sections as mutually illuminating, where one narrator’s discourse becomes a refracting surface for the other, is persuasive and offers a meaningful narratological intervention. The paper is at its best when it demonstrates how Bekim’s opening sexual encounter primes the reader’s reception of Emine’s later descriptions of masculinity and heterosexual marriage, producing a layered, dialogic effect. This is a strong formal claim that helps the manuscript avoid reducing the novel to a theme alone.

 A further strength lies in the manuscript’s willingness to foreground women’s positioning, especially Emine’s experiences of patriarchal violence, as not merely background to Bekim’s queer trajectory but as a central political concern. The argument that the novel is “not only an intervention in queer politics, but also in the politics of feminism and migration” is convincing, and it is one of the paper’s most ethically attentive gestures. By tracing how femininity is constructed as “not-men,” and how women’s labour and suffering are normalised through tradition, the article reminds readers that gender politics in the novel cannot be reduced to questions of sexual orientation or male desire.

 The paper’s framing of conservative and progressive readings is useful as an opening map, but it risks becoming too dominant as the analysis unfolds. At times, the manuscript appears to treat these positions as interpretive endpoints rather than provisional lenses. This can inadvertently flatten the novel’s ambivalence into a two-party debate. The author might strengthen the argument by clarifying that these are readerly tendencies rather than stable ideological camps, and by allowing more space for intermediate or non-aligned readings (such as postcolonial, disability/trauma-informed, affect-theoretical, or intersectional feminist approaches).

 Relatedly, the manuscript would benefit from a more explicit acknowledgement that “conservative” and “progressive” are culturally situated terms. A reader from the Balkans, the Nordic region, or the Middle East may not recognise themselves in these categories in the same way as an Anglophone academic audience might. A short methodological paragraph clarifying the geopolitical limits of these labels would make the framework feel more careful and less universalising.

 The manuscript occasionally employs phrases such as “healthy identity,” “abnormal,” or “wishy-washy gendered relativity.” Even though these terms are often attributed to a “folk psychology” viewpoint, they may still carry evaluative force that could alienate readers or create ambiguity about the author’s own stance. I would recommend either (a) placing such terms consistently in quotation marks and explicitly attributing them to a conservative interpretive position, or (b) replacing them with less value-laden alternatives (such as “socially intelligible identity,” “normatively legible identity,” “culturally sanctioned coherence”).

 Similarly, the argument that Bekim’s in-betweenness “stands in the way of a coherent or ‘healthy’ identity” may be read as pathologising unless it is very clearly framed as a description of how certain readers might interpret the character. This is especially important given the manuscript’s interest in how ideology shapes reading practices.

 The paper contains several excellent close readings, but the pacing sometimes slows due to long stretches of summary. This is most noticeable in the later sections where the narrative events are retold in detail. The author could strengthen the scholarly voice by condensing plot summaries and foregrounding interpretive claims more consistently. For instance, the discussion of Bekim’s relationship with the cat and its mirroring of Emine’s marriage is persuasive, but it could be streamlined so that each quoted passage is explicitly tied to the analytic stakes of sex/gender categorisation, practical identity, and readerly politics.

 Additionally, the manuscript sometimes introduces theoretical concepts (for example, implied reader, polychronic narration, Bakhtinian double voicing) in quick succession. While the scholarship is impressive, the density may overwhelm readers. A clearer signposting of how each theoretical tool is necessary for the argument would help. The author might consider prioritising fewer frameworks and developing them more fully, rather than assembling many concepts in parallel.

 The manuscript notes that race and immigrant experience are central themes in the novel, but it largely brackets them as “the work of another paper.” Given that the argument is about the politics of ambiguity, it may be worth integrating race and migration more directly, even briefly, because sex/gender ambiguity in Bekim’s case is inseparable from his ethnic positioning and his experience of being marked as an immigrant in Finland. The paper’s strongest moments already gesture toward this (for instance, shame, language, majority-culture Finns, and the cat’s racism). Expanding this dimension would prevent the analysis from appearing overly gender-focused in isolation.

 This integration would also help avoid the risk of presenting sex/gender politics as detachable from other axes of power. A short paragraph linking the novel’s queer politics to racialization and Nordic exceptionalism would add depth and align with the manuscript’s claim that multiple readings are invited.

 Offering some editorial suggestions, there are occasional typographical issues (“heuristically” should be “heuristically”) and small inconsistencies in punctuation and spacing. The manuscript sometimes shifts between “sex/gender” as a compound term and “sex and gender” as separate categories. It would help to define the term early and then use it consistently. Also, consider reducing footnote density or moving some explanatory footnotes into the main text where they are essential to the argument. Some quotations are very long; shortening them and offering more interpretive commentary would improve readability. The conclusion is thoughtful, but it could be slightly sharper in restating the paper’s unique contribution: namely, that ambiguity functions as a political structure of reading rather than a mere thematic feature.

 Overall, I find this manuscript to be a valuable and original contribution to the study of Statovci’s *My Cat Yugoslavia* and to broader debates about how narrative ambiguity produces ideological multiplicity. With moderate revision, particularly in refining the conservative/progressive heuristic, tightening structure, and clarifying terminology, the paper would be well-positioned for publication. I therefore recommend: Minor Revisions / Revise and Resubmit (depending on the journal’s standards).

 I appreciate the author’s careful work and the seriousness with which the manuscript engages both literary form and political interpretation. With revision, this piece has the potential to become a strong reference point for scholars working at the intersection of narratology, queer studies, and the politics of reading.

If any, are all the source data and materials underlying the results available?

No source data required

Is the work original in terms of material and argument?

Yes

Is the work clearly and cogently presented?

Partly

Is the argument persuasive and supported by evidence?

Partly

Does the research article contribute to the cultural, historical, social understanding of the field?

Yes

Does it sufficiently engage with relevant methodologies and secondary literature on the topic?

Yes

Reviewer Expertise:

Dr Alan Ali Saeed’s research focuses on British modernist women writers, particularly their use of stream-of-consciousness and philosophical narrative techniques. He also explores feminist autobiography as a form of resistance and identity formation. His work bridges literature with philosophy and psychology through interdisciplinary narrative analysis. He is increasingly interested in representations of Kurdish identity in fiction, as well as visual storytelling in graphic novels and film. Across all areas, his scholarship emphasises marginalised voices and gendered narrative strategies.

I confirm that I have read this submission and believe that I have an appropriate level of expertise to confirm that it is of an acceptable scientific standard.

# Reviewer report: Reviewer response for version 1

**DOI:** 10.21956/openreseurope.22493.r59450

**Published:** 2025-09-23

**Authors:** Ralph J. Poole

**Affiliations:** 1 University of Salzburg, Salzburg, Austria

The article offers an intriguing reading of Statovci’s novel, arguing persuasively for a politics of ambiguity and thereby delivering a great example of a politics of interpretation. The paper’s strength lies in demonstrating how the duality of narrators sheds light on the ways gender codes echo and mirror through transferences across the male and female narrators. The author uses Faust-Sterling’s “sex/gender”-argument, which, although a bit dated, remains valid and important to debunk normative assumptions about sex and gender binaries. Some of her most provocative claims about the multiplicity of sexes remain undiscussed, however, in this article, and I wonder if an understanding of Bekim’s character could not have been pushed a bit further towards a more progressive queerness. Emine’s character gets an important treatment; her voice is prominent, a relevant aspect of the politics of reading at work. Applying Warhol’s “doubleness” is also a good choice to show that binaries need not be simple opposites but can be complex and contradictory coexisting forces. While I can see the benefit of pitting a conservative against a progressive reading, I take issue with some claims regarding the readerly positioning. I like the strong voice and the clear first-person of the author, which makes me all the more wonder about statements such as “the sex/gender of the flesh-and-blood reader” or “The novel artfully puts male readers …”. As a queer reader myself, I ask for more nuance and the acknowledgment that I might read quite differently from the male writer of this article, especially in a paper that methodologically employs queer theory and feminist narratology. Also, another point regarding narratology is the question of genre: with talking animals and human-animal sexual relationships, the novel clearly diverges from realist settings. Does this help us understand the particular queerness of Bekim more or less? At what point and why does he “return” to human-only relationships, and what is gained or lost regarding his particular queerness? Would an alternative framework beyond the conservative/progressive binary be beneficial here (posthuman, e.g.)? Even though this might have been covered in other readings of the novel, the “fantastic” element might be foregrounded as emphasizing the surrealness of sex/gender identity politics.

If any, are all the source data and materials underlying the results available?

Yes

Is the work original in terms of material and argument?

Yes

Is the work clearly and cogently presented?

Yes

Is the argument persuasive and supported by evidence?

Yes

Does the research article contribute to the cultural, historical, social understanding of the field?

Yes

Does it sufficiently engage with relevant methodologies and secondary literature on the topic?

Yes

Reviewer Expertise:

gender studies, queer theory, literary studies

I confirm that I have read this submission and believe that I have an appropriate level of expertise to confirm that it is of an acceptable scientific standard, however I have significant reservations, as outlined above.

# Response

**Published:** 2026-01-13

**Authors:** Eric Bergman

**Affiliations:** Croatian studies, Sveuciliste u Zagrebu Filozofski fakultet, Zagreb, City of Zagreb, Croatia

Dear Professor Poole, Thank you for the valuable review! I really appreciate that you’ve taken time to give valuable feedback. This is the first time I’m working on ORE and there were some technical difficulties, so I’ve only now managed to get the paper re-uploaded and to respond to you. First, I understand that the ‘progressive’ end of the progressive/conservative continuum could be a more progressive based on, e.g., Faust-Sterling’s work. Point taken. As it was, the fullness of the continuum was not accounted for. In fact, the only way to approach accounting for it is to say outright that there is a spectrum of multiplicity (this was one of the central points in my dissertation) and this paper takes up a simplified heuristic based on normativity and ‘folk psychology,’ etc. I’ve added text indicating that these heuristic poles—and the biological binary of male/female sex—can be rather understood as a one part of a spectrum of multiplicity. The animals in the novel (see below) and a possible posthuman reading could mean that the “‘fantastic’ element might be foregrounded as emphasizing the surrealness of sex/gender identity politics,” as you wrote. I added that if the delimited (from the perspective of a spectrum of mulitplicity) option of male-male sex is seen as abnormal, then what about a cat or snake? It could be mocking the whole question of delimited options, which is “sex/gender identity politics,” as you say. As for my assumptions about the ‘flesh-and-blood-reader,’ you are totally right! This is annoying in any text, but especially in a text with a topic such as this. This is one of Warhol’s points precisely. I’ve opened that up beyond the limited heuristic used to frame the paper overall. These readings in no way account for the multiplicity of potential readings depending, in part, on the positionality of the reader. In terms of genre, much of the scholarly attention given to this novel so far in Finland (the novel is quite recent so there isn’t a whole lot, mostly in the form of theses) has deemed it ‘magical realism.’ I even found an interview in *The Guardian* where Statovci himself positions the work in this genre. “Foregrounding the surrealness of sex/gender identity politics” is a really succint way of interpreting what animals do in terms of the paper’s topic and very usefully expands the sex/gender heursitic into multiplicity. The handling of animals deserves more attention; for now, I’ve added ideas along the lines of what you suggested. In the paper, I argue that Bekim’s relations with animals coincides with his loneliness and angst; when he returns to a relation with Sami, he approaches something like contentment. (In the process, he kills the snake, which has all kinds of potential as a turning point in the narrative. The paper is very long as it is, and I think this should be taken up in another paper.) Thank you very much! I respect your openness and value your feedback. Eric

# Reviewer report: Reviewer response for version 1

**DOI:** 10.21956/openreseurope.22493.r59449

**Published:** 2025-09-19

**Authors:** John paolo Sarce

**Affiliations:** 1 Ateneo de Manila University, Quezon City, Metro Manila, Philippines

It would strengthen the paper if the ideas and concepts were first **clustered or organized** before being applied to the text. The introduction primarily foregrounds Fausto-Sterling’s work, but the article also draws on several other studies, which could be more effectively integrated if grouped thematically.

 Regarding the method, the idea of **double reading** is compelling. However, it would be more impactful if this concept were reiterated later in the paper or applied across different sections, rather than only being briefly mentioned as “doubleness.” Consistently using and developing this term would help create a more coherent and unified reading.

 Third, since the material heavily engages with **U.S.-based scholarship**, it would be valuable for the article to include a section contextualizing **queer Finnish literature** before analyzing *My Cat Yugoslavia*. This would ground the discussion locally and provide a richer foundation for the novel’s interpretation.

If any, are all the source data and materials underlying the results available?

Yes

Is the work original in terms of material and argument?

Yes

Is the work clearly and cogently presented?

Partly

Is the argument persuasive and supported by evidence?

Yes

Does the research article contribute to the cultural, historical, social understanding of the field?

Yes

Does it sufficiently engage with relevant methodologies and secondary literature on the topic?

Yes

Reviewer Expertise:

Queer theory and Literary studies, Teaching of Literature, Medical humanities, Digital Humanities, Cultural Studies

I confirm that I have read this submission and believe that I have an appropriate level of expertise to confirm that it is of an acceptable scientific standard.
